# Construction of a Three-Dimensional BaTiO_3_ Network for Enhanced Permittivity and Energy Storage of PVDF Composites

**DOI:** 10.3390/ma14133585

**Published:** 2021-06-27

**Authors:** Xueqing Bi, Lujia Yang, Zhen Wang, Yanhu Zhan, Shuangshuang Wang, Chunmei Zhang, Yuchao Li, Yinggang Miao, Junwei Zha

**Affiliations:** 1 School of Materials Science and Engineering, Liaocheng University, Liaocheng 252059, China; bixueqing@gmail.com (X.B.); Iujiay0582@yeah.net (L.Y.); 2010180105@stu.lcu.edu.cn (Z.W.); zhanyanhu@lcu.edu.cn (Y.Z.); wangshuangshuang@lcu.edu.cn (S.W.); zhangchunmei@lcu.edu.cn (C.Z.); 2 Joint International Research Laboratory of Impact Dynamics and Its Engineering Applications, School of Aeronautics, Northwestern Polytechnical University, Xi’an 710072, China; miaoyinggang@nwpu.edu.cn; 3 School of Chemistry and Biological Engineering, University of Science and Technology Beijing, Beijing 100083, China; zhajw@ustb.edu.cn

**Keywords:** poly(vinyl difluoride), barium titanate, dielectric constant, 3D network, energy storage

## Abstract

Three-dimensional BaTiO_3_ (3D BT)/polyvinylidene fluoride (PVDF) composite dielectrics were fabricated by inversely introducing PVDF solution into a continuous 3D BT network, which was simply constructed via the sol-gel method using a cleanroom wiper as a template. The effect of the 3D BT microstructure and content on the dielectric and energy storage properties of the composites were explored. The results showed that 3D BT with a well-connected continuous network and moderate grain sizes could be easily obtained by calcining a barium source containing a wiper template at 1100 °C for 3 h. The as-fabricated 3D BT/PVDF composites with 21.1 wt% content of 3D BT (3DBT–2) exhibited the best comprehensive dielectric and energy storage performances. An enhanced dielectric constant of 25.3 at 100 Hz, which was 2.8 times higher than that of pure PVDF and 1.4 times superior to the conventional nano–BT/PVDF 25 wt% system, was achieved in addition with a low dielectric loss of 0.057 and a moderate dielectric breakdown strength of 73.8 kV·mm^−1^. In addition, the composite of 3DBT–2 exhibited the highest discharge energy density of 1.6 × 10^−3^ J·cm^−3^ under 3 kV·mm^−1^, which was nearly 4.5 times higher than that of neat PVDF.

## 1. Introduction

Flexible polymer dielectrics with fast charge–discharge speeds and a high-power density exhibit advantages over many batteries and supercapacitors, particularly in the field of 5G/6G technologies, electromagnetic weapons, power engineering, and other microelectronic devices [1,2,3]. However, the low permittivity and poor energy storage density of polymers limit their vast industrial applications. Among the well-known polymers, poly(vinyl difluoride) (PVDF) and its derivates, possessing a relatively high dielectric response and flexibility show potential applications in dielectric storage and conversion. Consequently, various studies have been reported on enhancing the permittivity and reducing the dielectric loss of PVDF-based composites; thus, meeting the requirements of high energy storage purposes [4]. From the energy storage equation of a dielectric material: U = 1/2ε_0_ε_r_E_b_^2^ [5], where E_b_ is the breakdown strength, and ε_0_ and ε_r_ are the vacuum and relative dielectric constant of the material, it is known that the increase in E_b_ and ε_r_ can effectively improve the energy storage capacity of materials. Nevertheless, simultaneously enhancing both ε and E_b_ is a technical bottleneck because a large permittivity usually results in a large dielectric loss, thus leading to the reduced breakdown strength and lifetime of electronic devices. Therefore, increasing the permittivity and maintaining breakdown strength play an important role in enhancing the overall energy storage capacity of composites.

Polymers incorporated with ferroelectric ceramic nanoparticles, such as barium titanate (BaTiO_3_, BT), lead zirconate titanate (PZT), barium strontium titanate (BST), etc., have been widely applied to improve the dielectric performances of polymer systems as these ceramics usually possess a high permittivity and a relatively low dielectric loss [6,7,8]. Additionally, the dielectric properties of polymer/ceramic composites can be well tailored by controlling the filler concentration as well as their microstructures. Hu et al. [6] prepared BST/PVDF composites using a solution casting method and found that the dielectric constant of the BST/PVDF composite with 40 vol% BST reached as high as 40 (1 kHz), together with a very low dielectric loss tangent (tanδ = 0.17) and an improved energy density (U = 0.36 J·cm^−3^). Luo et al. [7] further improved the dielectric performances of BT/PVDF systems by depositing Ag nanoparticles onto the surface of BT. A high dielectric constant of 94.3 and low loss of 0.06 were achieved for BT@Ag/PVDF composites with 43.4 vol% BT@Ag content (1 kHz). The presence of conducting nano-Ag effectively promoted the interfacial polarization of PVDF while not forming a conductive network, implying promising applications in electronic devices.

Higher filler loading usually results in a poor flexibility of polymer dielectrics, thus leading to poor stability and processability. Accordingly, structural design is inevitable in obtaining a comprehensive dielectric performance of composites. Xie et al. [8] introduced core–shell ployimide@BaTiO_3_ (PI@BT) filler into a PVDF matrix. The core–shell structured PI@BT, for one thing, enhanced the interfacial polarization between the filler and matrix, and, for another, effectively limited the charge leakage, thus improving the dielectric breakdown strength as well. Wang et al. [9] designed a sandwich structure by using PVDF as the intermediate layer and BT/PVDF as the outer layers. An ultrahigh energy density of 16.2 J·cm^−3^ was obtained at a breakdown strength of 410 kV·mm^−1^. Luo et al. [10] presented a new strategy by constructing a three-dimensional (3D) BT network in epoxy resin. The dielectric constant of the composite reached 200 with a relatively low filler loading of 30 vol%. In addition to this, the energy density exhibited was 16 times higher than that of neat epoxy matrix.

In this work, a novel continuous 3D BT network is easily constructed by calcinating a cleanroom wiper template containing barium resource gelation. The inexpensive cleanroom wiper composed of 45 wt% polyester fiber and 55 wt% cellulose, exhibiting an interwoven fiber network with a high adsorption ability. These allowed the construction of a 3D BT network completely along the orientation of the wiper fiber filaments. The 3D BT/PVDF dielectric composites were then fabricated by inversely introducing a PVDF solution into the above-mentioned 3D BT network. The 3D BT network was expected to provide a continuous polarization pathway, thus effectively increasing the dielectric constant of the composites at a small filler loading. The effect of microstructure and content of the 3D BT on the dielectric and energy storage performances were systematically studied.

## 2. Materials and Methods

### 2.1. Materials

Barium acetate, diacetone, and tetrabutyl titanate were supplied by Tianjin Kermel Chemical Reagent Co. Ltd. (Tianjin, China) and were used as received. Ethylene glycol methyl ether, acetic acid glacial, N-methyl pyrrolidone and N,N-dimethylformamide (DMF) were purchased from Sigma Aldrich Company. Poly(vinylidene fluoride) (PVDF, Kynar 740) was supplied by Atofina Chemicals Inc. (Houston, TX, USA). Barium titanate nanoparticles (nano–BT, diameter = 500 nm, 99.9% metals basis) were obtained from Ourchem Company (Guangzhou, China). Cleanroom wiper (45 wt% polyester fiber and 55 wt% cellulose, areal density is 135 g·m^−2^, thickness is ~100 μm) was purchased from Wuchenbu factory, Kunshan, Jiangsu, China [11].

### 2.2. Preparation of 3D BT Network

**Figure 1:**
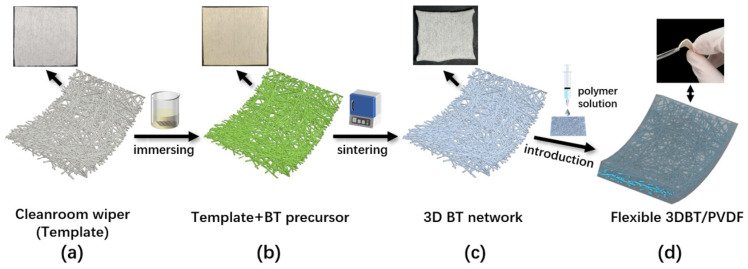
Schematic illustration of the fabrication of 3D BT/PVDF composites. The upper-left displays digital images of each process.

The fabrication of the 3D BT network was based on the sol-gel method. More visualized schematic procedures are shown in Figure 1. Briefly, 12.77 g of barium acetate was dissolved in 30 mL acetic acid (AR, ≥99.5) at 80 °C. The barium solution was then cooled down to room temperature and followed by the addition of 52.3 mL of ethylene glycol methyl ether, 17.6 mL tetrabutyl titanate, and a little acetylacetone (0.1 mL) to obtain 0.5 M of barium titanate (BaTiO_3_) precursor. The cleanroom wiper templates (Figure 1a) with a dimension of 40 × 40 × 0.1 mm^3^ were cut from one piece of the received cleanroom wiper and immersed in the above–mentioned precursor solution for 2 h with intermittent ultrasonication. The barium precursor was then infiltrated into the porous wiper template until saturation. After drying in an oven (60 °C) to eliminate the acetylacetone, the barium precursor solution rapidly became a gelation (Figure 1b). For comparison, the saturated template was placed in a muffle oven and sintered at different temperatures (1000 °C, 1100 °C, and 1200 °C) for three hours at a heating rate of 4 °C·min^−1^ under an air atmosphere. Finally, the BT with a continuous three-dimensional network, similar to the wiper’s interwoven structure, was constructed (Figure 1c).

### 2.3. Preparation of 3D BT/PVDF Composites

PVDF was dissolved in a solution mixture of DMF and acetone (molar ratio of 1:1) to obtain a 10 wt% PVDF solution. A small amount of the PVDF solution (2 mL, 1.5 mL, 1 mL) was then introduced dropwise into the above-mentioned 3D BT network (Figure 1d). After infiltration, flexible 3D BT/PVDF composites were finally obtained after removing the solvent in an oven at 80 °C, and they were denoted as 3DBT–1, 3DBT–2, and 3DBT–3, respectively. In comparison, conventional nano–BT/PVDF composites with contents of 25 wt% and 50 wt% were prepared by adding 0.94 g and 2.81 g of nano–BT to 30 mL of the above-mentioned PVDF solution (10 wt%), and were designated as BT–1 and BT–2, respectively. The mixtures were then cast onto a glass plate (30 cm × 20 cm) using laboratory casting equipment (MSK-AFA-III, Hefei Kejing Co., Ltd.) with a casting thickness of 100 μm. Table 1 describes the composition and designation of the 3D BT/PVDF composites. The contents of the 3D BT were obtained using TGA and the results are in Figure 9. The volume percentage of BT was calculated using Equation (1), where the density of BT (*ρ*_1_) and PVDF (*ρ*_2_) are 5.85 g·cm^−3^ and 1.78 g·cm^−3^, respectively [12].

(1) $$vol\%=\frac{wt\%/\rho_{1}}{wt\%/\rho_{1}+\left(1-wt\%\right)/\rho_{2}}$$

### 2.4. Characterization

The crystalline structure of the 3D BT and the microstructure of the composites were examined using an X-ray diffractometer (XRD, D8 ADVANCE, Bruker, Karisruhe, Germany) in the range of 5–80° with a scanning rate of 6°·min^−1^. The morphologies of the 3D BT network and the 3D BT/PVDF composites were observed using a field emission electron microscope (FESEM, Zeiss Merlin Compact) and an optical microscope (CX43, Olympus, Tokyo, Japan). The thermal property was determined on a synchronous thermal analyzer (STA 449C, Netzsch, Bavaria, Germany) from 20 °C to 700 °C with a heating rate of 10 K·min^−1^ under an oxygen atmosphere. A Precision LCR Meter (UC2876, Ucetech, Changzhou, China) was used to measure the dielectric and electrical properties of the materials within a frequency range from 50 Hz to 5 MHz. To eliminate surface resistance, both sides of the composite samples were coated with a self-made thin layer of carbon black-based conducting adhesive (carbon black 8 wt%/epoxy mixture, electrical conductivity >0.2 S·m^−1^) as electrodes. The diameter of each electrode was 4 mm. All specimens were measured at least 5 times for accuracy. The breakdown strength of the materials was obtained via a dielectric strength tester (ZJC–50KV, Beijing Air Times, Beijing, China) with a voltage rise of 1 kV·s^−1^.

## 3. Results and Discussions

### 3.1. Structure and Morphology of 3D BT Network

**Figure 2:**
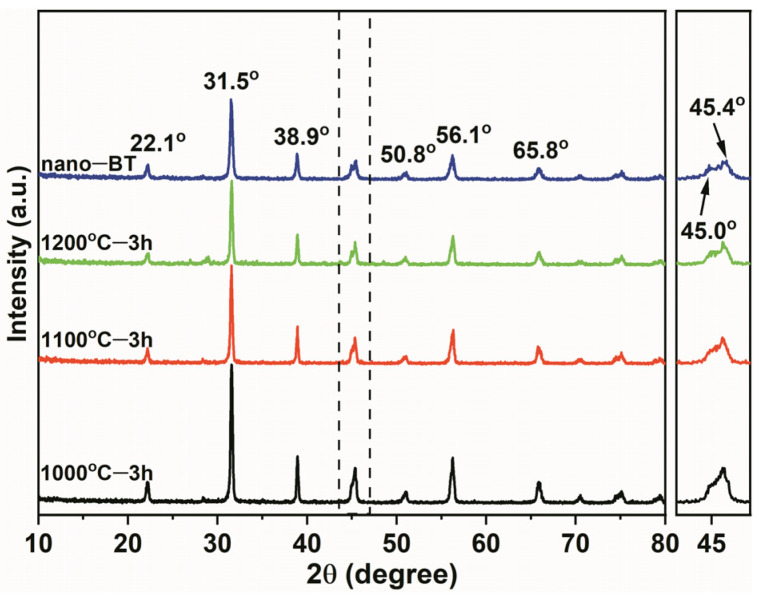
XRD patterns of 3D BT ceramics sintered at 1000 °C, 1100 °C, and 1200 °C and nano–BT.

Figure 2 displays the XRD patterns of the 3D BT (BaTiO_3_) ceramics sintered at 1000 °C, 1100 °C, and 1200 °C and the nano–BT, respectively. The distinct peaks at 2θ = 22.1°, 31.5°, 38.9°, 45.4°, 50.8°, 56.1°, and 65.8° correspond to the (100), (110), (111), (200), (210), (211), and (220) characteristic diffractions of cubic BT, respectively. As compared with the XRD curve of the cleanroom wiper in Figure S1a (Supplementary Materials), the characteristic peaks of the cleanroom wiper were diminished after sintering due to the thorough decomposition of the cleanroom wiper at high temperature, instead of the BT ceramic characterization peaks. The TGA curve in Figure S1b further verifies the full decomposition of the cleanroom wiper under an oxygen environment. The initial decomposition at 5% weight loss occurred at 283.5 °C. After 458 °C, most of the cleanroom wiper was degraded and there was almost nothing left at 700 °C. Furthermore, it was seen from the enlarged XRD peak in the vicinity of 45° that the intensity of the (200) peak at 45.4° decreased gradually with increasing sintering temperature. In the meanwhile, a distinct increase of the peak at 45.0° could be observed, which is corresponded to the (002) peak of the tetragonal BT [13], indicating a cubic to tetragonal transition of the BT crystal. It is well known that the tetragonal type of BT contributes a higher permittivity than that of the cubic one [14].

**Figure 3:**
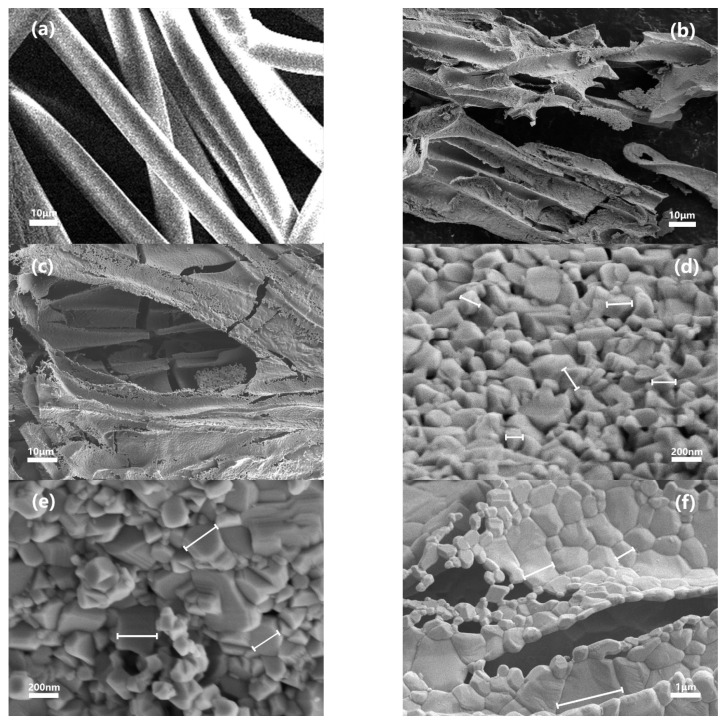
SEM images of (**a**) cleanroom wiper, 3D network structure BT sintered at (**b**) 1000 °C—3 h and (**c**) 1100 °C—3 h, and higher magnification images of 3D BT network structure obtained at (**d**) 1000 °C—3 h, (**e**) 1100 °C—3 h and (**f**) 1200 °C—3 h.

Figure 3 displays representative SEM images of the cleanroom wipers and the 3D BT network structure. The cleanroom wiper exhibited a porous interwoven structure (Figure 3a), which provided a 3D skeleton for the formation of the 3D continuous BT network (Figure 3b,c). Furthermore, it is seen that the microstructure of the 3D BT sintered at 1000 °C (Figure 3d) possessed a smaller and more uniform grain size than those 3D BT sintered at 1100 °C (Figure 3e) and 1200 °C (Figure 3f). However, abnormal grain growth can be clearly observed at a high sintering temperature of 1200 °C, resulting in a larger and ununiform grain size of 1–3 μm (Figure 3f). Therefore, in this study, the 3D BT used for fabrication of the following 3D BT/PVDF composites was sintered at 1100 °C as it possessed a much better continuous network structure with uniform small grain sizes and, of course, more tetragonal forms of BT.

### 3.2. Structure and Morphology of 3D BT/PVDF Composites

**Figure 4:**
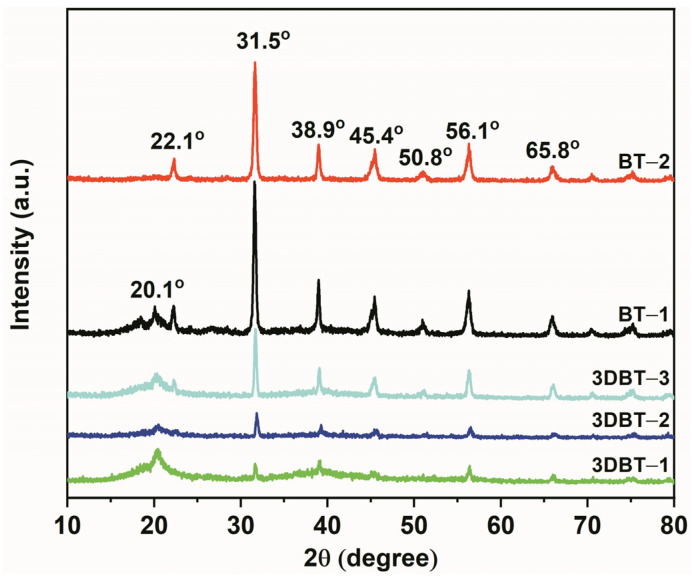
XRD patterns of the nano–BT/PVDF and 3D BT/PVDF composites.

Figure 4 illustrates the XRD patterns of the 3D BT/PVDF and nano–BT/PVDF systems. The wide peak at 2θ = 20.5° is the characteristic diffraction peak of α-PVDF. Other peaks located at 22.1°, 31.5°, 45.4°, 50.8°, 56.1°, and 65.8° are the characteristic peaks of BT. With the increase in BT content, the peak of the PVDF at 20.1° gradually decreases and almost disappears at 50 wt% BT loading (BT–2), which is due to the fact that the addition of ceramic filler destroys the ordered arrangement of the PVDF molecules [15].

**Figure 5:**
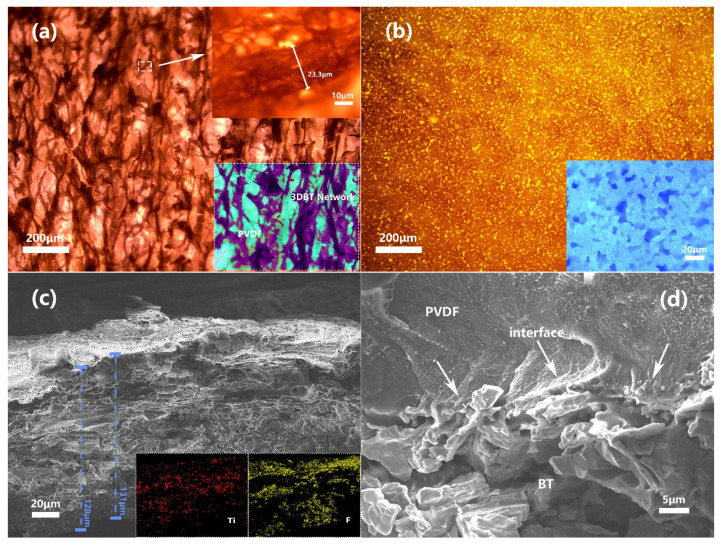
Representative optical microscopes of (**a**) 3DBT–2 and (**b**) BT–1 composites, and (**c**) low and (**d**) high magnification of SEM images of 3DBT–2 system.

The microstructure and morphology of the 3D BT/PVDF system is shown in Figure 5. Figure 5a,b shows representative optical microscope images of 3DBT–2 and BT–1, respectively. An interwoven structure can be clearly observed in the 3DBT–2 composite (Figure 5a) as compared with that of the BT–1 system (Figure 5b), indicating the successful formation of a continuous 3D BT network. We also present a deep contrast color image in the localized lower-right area in Figure 5a. The purple vein further indicates a well-connected 3D continuous network structure. The inset in the top right of Figure 5a displays an enlarged BT filament with a diameter of 23.3 μm, which is a little larger than that of original wiper fibers, as shown in Figure 3a. Figure 5c,d shows the high and low magnification SEM images of the 3DBT–2 system. It can be seen from the cross section of the 3DBT–2 system and the insets of the EDS (F, Ti) element analyses (Figure 5c) that the PVDF matrix is successfully infiltrated into the 3D BT network. The thickness of the 3D BT and its PVDF composites is about 130 μm, as determined form the SEM images. Figure 5d indicates that the PVDF matrix has a relatively good interfacial adhesion and compatibility with the BT filaments. The good adhesion between the PVDF matrix and the BT may be ascribed to the large specific surface area and the curved structure of the 3D BT, which possesses more surface energy and a large adsorption ability. The presence of the 3D BT network is expected to further provide a continuous polarization channel and a uniform electric field [16], thus improving the dielectric and energy storage performances of the composites.

### 3.3. Electrical Properties

**Figure 6:**
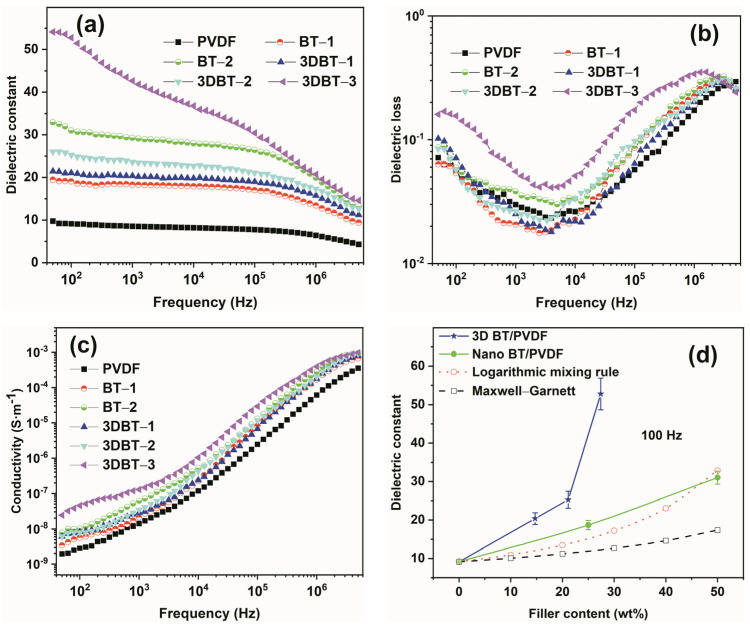
Frequency dependence of (**a**) dielectric constant, (**b**) loss tangent, (**c**) conductivity of 3D BT/PVDF composites, and (**d**) the dielectric constant as a function of filler contents.

Figure 6a–c displays the frequency spectra of dielectric constant, dielectric loss, and conductivity, respectively. It is seen from Figure 6a that the permittivity of all composites decreases gradually with increasing frequency, indicating the hysteresis of polarization of composites with external frequencies [17]. As expected, the dielectric constant increases with increasing 3D BT and BT contents, implying an enhanced interfacial polarization after the addition of nanofillers. However, the dielectric constant for the fabricated 3D BT/PVDF composites increases more distinctly than that of the nano–BT/PVDF system in the whole frequency range, indicating a larger polarization effect in the presence of the 3D connected network. Figure 6b exhibits the variation in the loss tangent with the external frequencies. It can be seen that the tanδ of the composites also increases with different filler loadings, particularly at a lower frequency range. A significant increase in tanδ can be observed in the 3DBT–3 system, which is mainly attributed to the large content of the 3D BT network, providing more pathway for carrier movement. The conductivity of the 3D BT/PVDF composites shown in Figure 6c increase almost linearly in log–log plots within the tested frequency range [18]. No frequency-independent plateau region of conductivity at low frequency is observed, implying a good insulating behavior for these composites. The variations in the dielectric constant of the 3D BT/PVDF and the nano–BT/PVDF systems with different filler loadings under 100 Hz is illustrated in Figure 6d. The dielectric constant of the pure PVDF is only 9.1 at 100 Hz and has a low loss tangent of 0.056. However, the dielectric constant of 3DBT–3 (27.4 wt%) composite reaches 52.8 (tanδ = 0.156), which is nearly six times higher than that of the neat PVDF. Furthermore, the permittivity is much higher than that of the traditional nano–BT/PVDF composite (ε = 31.0) with 50 wt% BT loading. The large permittivity with a relatively low 3D BT loading is mainly attributed to the constructed 3D network, which provides a continuous polarization channel throughout the whole sample [19]. In addition, the experimental results of the nanocomposites are also compared with those predicted theoretical models [20]. This reveals that the logarithmic mixing expression can well describe the dielectric response of the PVDF composites filled with BT nanoparticles. However, both the logarithmic mixing rule and Maxwell–Garnett approximation cannot predict the dielectric response of the 3D BT/PVDF systems because the BT filler is no longer spherical when forming a 3D interwoven structure. The dielectric constant and loss tangent of each composite are summarized in Table 2.

**Figure 7:**
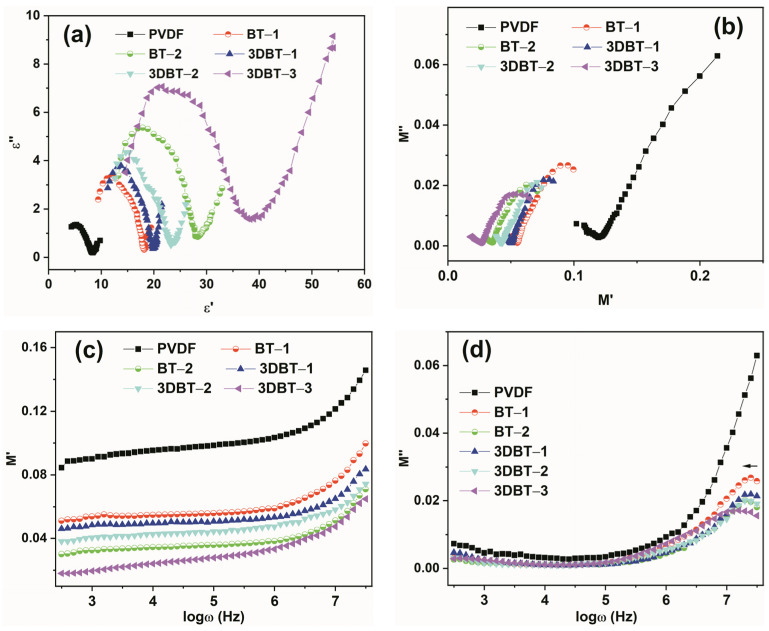
The variation of (**a**) ε’–ε” and (**b**) M’–M” (dielectric modulus) and frequency dependence of the (**c**) real and (**d**) imaginary part of dielectric modulus.

Figure 7a,b show the ε’–ε” and M’–M” (dielectric modulus) plots, respectively, showing different relaxation behaviors of the 3D BT/PVDF and nano–BT/PVDF systems [21]. It is clearly observed that the ε’–ε” plots (Figure 7a) of PVDF and its composites are no longer classical semicircles. An arc-like relaxation only occurs at a higher frequency and the diameters of these arcs increase with increasing BT and 3D BT contents. Accordingly, these arise from two polarization mechanisms. The first originates from dipole relaxation at higher frequencies, while the latter is attributed to the interfacial polarization at lower frequencies due to the presence of inorganic nanofillers [22]. The variation of M’ to M” also illustrates two relaxation behaviors, which exhibit a reverse trend to the ε’–ε” plots. In addition, from the frequency dependence of the dielectric modulus (Figure 7c,d), it can be observed that the addition of inorganic fillers, particularly the formation of the 3D BT network, can distinctively suppress the relaxation peak to lower frequencies [23]. The modulus formalism is particularly useful to show the relaxation behavior of PVDF and its composites.

### 3.4. Breakdown Strength and Energy Storage

The breakdown strength was determined using a two-parameter Weibull statistical distribution method [24], which can be written as Equation (2):

(2) $$P=\exp\left[-{\left(\frac{E_{b}}{E_{0}}\right)}^{\beta }\right]$$

where *E_b_* is the experimental breakdown strength, *P* is the cumulative probability of electrical failure, *β* is the shape parameter, which is related to the scatter of the data, *E*_0_ is the scale parameter, which represents the breakdown strength at the cumulative failure probability of 63.2%. Equation (2) can be derived into the logarithm Equation (3), where $\ln\left[-\ln\left(1-P\left(E_{b}\right)\right)\right]$ shows a linear relationship with $\ln E_{b}$.

(3) $$\ln\left[-\ln\left(1-P\left(E_{b}\right)\right)\right]=\beta lnE_{b}-\beta lnE_{0}$$

**Figure 8:**
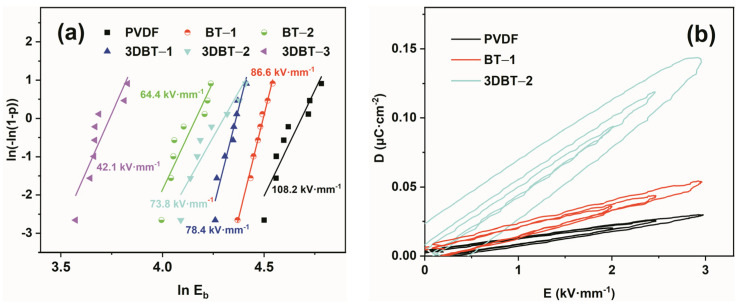
(**a**) Weibull plots for breakdown strength of the BT–1, BT–2, 3DBT–1, 3DBT–2, 3DBT–3, and neat PVDF, (**b**) unipolar D–E hysteresis loops of representative PVDF, BT–1, and 3DBT–2.

Figure 8a displays the Weibull distribution of breakdown strength. It is seen that the *E_b_* of the 3D BT/PVDF composites decreases more sharply than that of the conventional nano–BT/PVDF system, implying the strong effect of constructing such a 3D BT network. The derived *E*_0_ for the 3DBT–2 (21.1 wt%) system is 73.8 kV·mm^−1^, which is 68.2% pure PVDF matrix (108.2 kV·mm^−1^). However, we cannot simply determine the energy storage behavior just from the reduced breakdown strength according to the energy storage theory. In this regard, the energy storage capacity of these dielectric materials was evaluated using a unipolar D–E analyzer under the same field strength (Figure 8b and Figure S2). Figure 8b show representative D–E loops of the PVDF, BT–1, and 3DBT–2 under different applied field strengths of 2.0, 2.5, and 3.0 kV·mm^−1^, respectively, as BT–1 and 3DBT–2 exhibit the best energy storage performance in their systems (Table 2). It can be observed that the 3DBT–2 system has the greatest electric displacements (D_max_), as high as ~0.143 μC·cm^−2^ at 3 kV·mm^−1^, which is nearly 2.7 times superior to the BT–1 composite, indicating the enhanced polarization of the composites filled with a continuous ceramic network. The energy storage density is calculated from D–E loops based on the integral U = ∫EdD [25]. The achieved U under 3 kV·mm^−1^ for the BT–1 and 3DBT–2 composites are 0.68 × 10^−3^ J·cm^−3^ and 1.6 × 10^−3^ J·cm^−3^, respectively (summarized in Table 2). The results suggest that the composite of the 3DBT–2 system exhibit the best improvement in terms of discharged energy density, which is 4.5 times higher than that of the neat PVDF (0.36 × 10^−3^ J·cm^−3^). A higher 3D BT content (i.e., 3DBT–3) leads to a reduced energy storage capacity because of the large dielectric loss, as discussed above. Table S1 summarizes the dielectric and energy storage properties of various BT-filled polymer dielectrics. It can be seen from the ratio of U/U_m_ that the values in this work are better than those of other reports, providing a new approach in achieving high energy storage polymer dielectrics.

### 3.5. Thermal Stability

**Figure 9:**
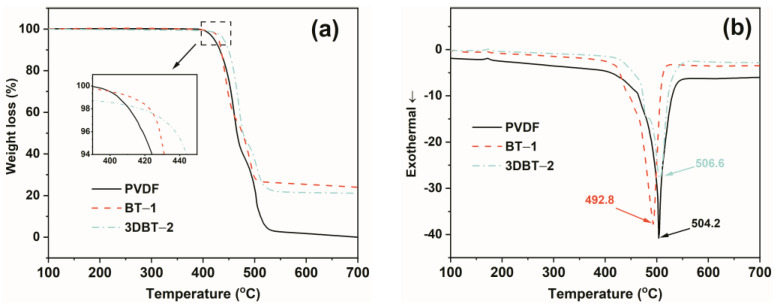
(**a**) TGA and (**b**) DSC curves of PVDF, BT–1, and 3DBT–2.

The thermal stability of representative PVDF, BT–1, and 3DBT–2 are illustrated in Figure 9. It can be seen from the TGA curves of Figure 9a that the initial decomposition temperature (T_d_, at 5 wt% weight loss) of the composites increases with the addition of inorganic ceramic fillers, particularly for the 3D BT system, implying enhanced thermal stability after the formation of the 3D network (Table 2). The introduction of the 3D BT network also improves the final decomposition temperature of the composites, as revealed by the DSC curves in Figure 9b. The final decomposition temperature of the 3DBT–2 increases by 2.4 °C compared to that of neat PVDF.

**Figure 10:**
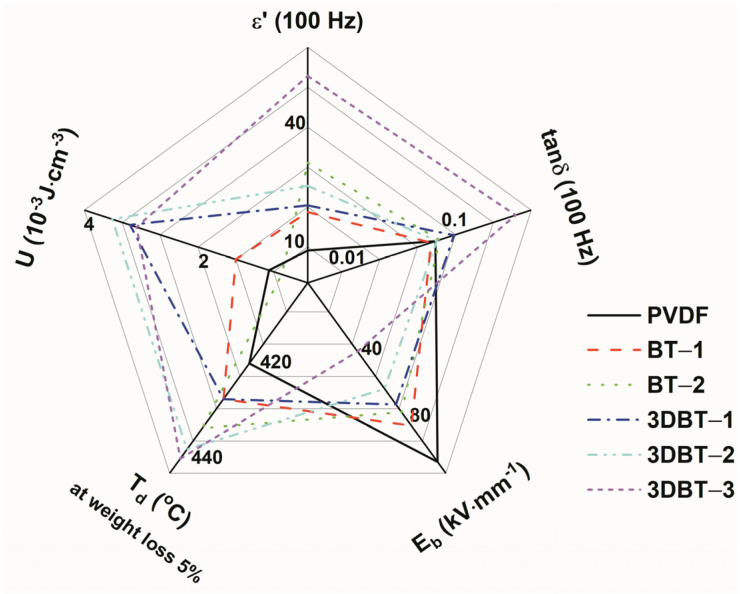
Dielectric, thermal and discharge energy storage performances of 3D BT/PVDF and nano–BT/PVDF composites.

Since it is arbitrary to judge a dielectric composite with high performance just by comparing the permittivity, dielectric loss, energy storage density, and breakdown strength because some of these parameters are contrary to each other, a star chart is presented in Figure 10, based on the above-mentioned representative results of PVDF, nano–BT/PVDF, and 3D BT/PVDF systems (Table 2) in order to have a macroscopic comparison of the composites. It is concluded that the 3D BT/PVDF composites possess comprehensive dielectric performances. The large energy density of the 3DBT–2 system is, of course, the concession of the dielectric constant, loss tangent, and breakdown strength. Essentially, the constructed 3D BT interwoven network plays an important role in enhancing the permittivity, reducing the dielectric loss, and maintaining the relatively good breakdown strength.

## 4. Conclusions

A novel three-dimensional ceramic network/polymer dielectric composite was successfully fabricated by inversely diluting PVDF solution into a 3D BT network. The 3D interwoven BT structure, not only provides continuous pathways for polarization, but also promotes the thermal stability of the composites. Consequently, the PVDF filled with 21.1 wt% 3D BT composite (3DBT–2) possesses the best energy storage capacity with a relatively high dielectric constant (25.3 at 100 Hz), low dielectric loss (0.057 at 100 Hz), and pertinent breakdown strength (73.8 kV·mm^−1^). The energy storage density of 3DBT–2 is 4.5 times higher than that of neat PVDF. Therefore, the design of a 3D BT network using a much cheaper cleanroom wiper provides a new approach in obtaining high energy storage polymer composites.

## Figures and Tables

**Table 1:** Composition and designation of conventional nano–BT/PVDF composites and the 3D BT/PVDF dielectric composites.

Composites	Designation	Content
3D BT/PVDF (2 mL)	3DBT–1	14.7 wt% (5.0 vol%) 3D BT
3D BT/PVDF (1.5 mL)	3DBT–2	21.1 wt% (7.5 vol%) 3D BT
3D BT/PVDF (1 mL)	3DBT–3	27.4 wt% (10.3 vol%) 3D BT
nano–BT (0.94g)/PVDF (30 mL)	BT–1	25 wt% (9.2 vol%) nano–BT
nano–BT (2.81g)/PVDF (30 mL)	BT–2	50 wt% (23.3 vol%) nano–BT

**Table 2:** Dielectric and thermal properties of PVDF composites.

Sample	ε’ (100 Hz)	tanδ (100 Hz)	E_b_ (kV·mm^−1^)	U (10^−3^ J·cm^−3^) at 3 kV·mm^−1^	T_d_ (5 % Weight Loss)
PVDF	9.1 ± 0.2	0.056 ± 0.02	108.2	0.359 ± 0.02	421.9
BT–1	18.7 ± 1.2	0.053 ± 0.003	86.6	0.676 ± 0.03	429.4
BT–2	31 ± 1.7	0.059 ± 0.003	64.4	0.272 ± 0.02	435.5
3DBT–1	20.4 ± 1.5	0.071 ± 0.007	78.4	1.566 ± 0.08	429.4
3DBT–2	25.3 ± 2.2	0.057 ± 0.006	73.8	1.604 ± 0.03	440.0
3DBT–3	52.8 ± 4.7	0.156 ± 0.006	42.1	1.469 ± 0.04	442.0

## Data Availability

Data is contained within the article.

## Supplementary Materials

The following are available online at https://www.mdpi.com/article/10.3390/ma14133585/s1, Figure S1: (a) XRD result and (b) Thermal performance of cleanroom wiper, Figure S2: Unipolar D–E hysteresis loops of representative PVDF, BT–1, BT–2, 3DBT–1, 3DBT–2, and 3DBT–3, respectively, Table S1: Dielectric and energy storage properties of the BT/polymer composites.

## References

[1] Kim H., Johnson J., Chavez L.A., Rosales C.A.G., Tseng T.-L.B., Lin Y. (2018). Enhanced Dielectric Properties of Three Phase Dielectric MWCNTs/BaTiO3/PVDF Nanocomposites for Energy Storage Using Fused Deposition Modeling 3D Printing. Ceram. Int..

[2] Dang Z.-M., Zheng M.-S., Zha J.-W. (2016). 1D/2D Carbon Nanomaterial-Polymer Dielectric Composites With High Permittivity for Power Energy Storage Applications. Small.

[3] Bao Z., Hou C., Shen Z., Sun H., Zhang G., Luo Z., Dai Z., Wang C., Chen X., Li L. (2020). Negatively Charged Nanosheets Significantly Enhance the Energy-Storage Capability of Polymer-Based Nanocomposites. Adv. Mater..

[4] Zhu L. (2014). Exploring Strategies for High Dielectric Constant and Low Loss Polymer Dielectrics. J. Phys. Chem. Lett..

[5] Liab Y., Bia X., Wanga S., Zhana Y., Liub H.-Y., Maib Y.-W., Liaoc C., Luc Z., Liaod Y. (2020). Core-Shell Structured Polyethylene Glycol Functionalized Graphene for Energy-Storage Polymer Dielectrics: Combined Mechanical and Dielectric Performances. Compos. Sci. Technol..

[6] Hu G., Gao F., Kong J., Yang S., Zhang Q., Liu Z., Zhang Y., Sun H. (2015). Preparation and Dielectric Properties of poly(vinylidene fluoride)/Ba0.6Sr0.4TiO3 Composites. J. Alloys Compd..

[7] Luo S., Yu S., Sun R., Wong C.-P. (2014). Nano Ag-Deposited BaTiO3 Hybrid Particles as Fillers for Polymeric Dielectric Composites: Toward High Dielectric Constant and Suppressed Loss. ACS Appl. Mater. Interfaces.

[8] Xie X., Yang C., Qi X.-D., Yang J.-H., Zhou Z.-W., Wang Y. (2019). Constructing Polymeric Interlayer With Dual Effects Toward High Dielectric Constant and Low Dielectric Loss. Chem. Eng. J..

[9] Wang Y., Cui J., Wang L., Yuan Q., Niu Y., Chen J., Wang Q., Wang H. (2017). Compositional Tailoring Effect on Electric Field Distribution for Significantly Enhanced Breakdown Strength and Restrained Conductive Loss in Sandwich-Structured ceramic/Polymer Nanocomposites. J. Mater. Chem. A.

[10] Luo S., Shen Y., Yu S., Wan Y., Liao W.-H., Sun R., Wong C.-P. (2016). Construction of a 3D-BaTiO3 Network Leading to Significantly Enhanced Dielectric Permittivity and Energy Storage Density of Polymer Composites. Energy Environ. Sci..

[11] Fu X., Li Y., Liao C., Gong W., Yang M., Li R.K.Y., Tjong S.C., Lu Z. (2019). Enhanced Electrochemical Performance of Solid PEO/LiClO4 Electrolytes With a 3D Porous Li6.28La3Zr2Al0.24O12 Network. Compos. Sci. Technol..

[12] Li Y., Tjong S.C. (2011). Structure and Electrical Characteristics of poly(vinylidene Fluoride) Filled With Beta Silicon Carbide Nanoparticles. J. Nanosci. Nanotechnol..

[13] Mallick S., Ahmad Z., Qadir K., Rehman A., Shakoor R., Touati F., Al-Muhtaseb S. (2020). Effect of BaTiO3 on the Sensing Properties of PVDF Composite-Based Capacitive Humidity Sensors. Ceram. Int..

[14] Chen C., Hao H., Wang T., Cheng J., Luo Z., Zhang L., Cao M., Yao Z., Liu H. (2019). Nano-BaTiO3 Phase Transition Behavior in Coated BaTiO_3_-Based Dielectric Ceramics. Ceram. Int..

[15] Qiu K., Yan H., Zhang D., Lu Y., Cheng J., Lu M., Wang C., Zhang Y., Liu X., Luo Y. (2014). Hierarchical 3D Co3O4@MnO2 core/Shell Nanoconch Arrays on Ni Foam for Enhanced Electrochemical Performance. J. Solid State Electrochem..

[16] Tang H., Zhou Z., Sodano H.A. (2014). Relationship Between BaTiO3 Nanowire Aspect Ratio and the Dielectric Permittivity of Nanocomposites. ACS Appl. Mater. Interfaces.

[17] Raghuram N., Rao T.S., Naidu K.C.B. (2020). Electrical and Impedance Spectroscopy Properties of Hydrothermally Synthesized Ba0.2Sr0.8-yLayFe12O19 (y = 0.2–0.8) Nanorods. Ceram. Int..

[18] Chaves A., Azadani J.G., Alsalman H., Da Costa D.R., Frisenda R., Chaves A.J., Song S.H., Kim Y.D., He D., Zhou J. (2020). Bandgap engineering of two-dimensional semiconductor materials. npj 2D Mater. Appl..

[19] Panomsuwan G., Manuspiya H. (2020). Dielectric Properties and Discharge Energy Density of Epoxy Composites With 3D BaTiO_3_ Network Structure. Mater. Lett..

[20] Hayashida K. (2016). Highly Improved Dielectric Properties of polymer/α-Fe2O3composites at Elevated Temperatures. RSC Adv..

[21] Das A., Hatada R., Ensinger W., Flege S., Baba K., Meikap A. (2018). Dielectric Constant, AC Conductivity and Impedance Spectroscopy of Zinc-Containing Diamond-Like Carbon Film UV Photodetector. J. Alloys Compd..

[22] Tong H., Liu X.-J., Zheng M.-S., Dang Z.-M., Zha J.-W. (2020). Dual Functionalized Janus Structural PVDF Nanocomposite with Surface-Modified Dielectric and Magnetic Nanoparticles. Appl. Phys. Lett..

[23] Tantis I., Psarras G.C., Tasis D. (2012). Functionalized Graphene—Poly(vinyl Alcohol) Nanocomposites: Physical and Dielectric Properties. Express Polym. Lett..

[24] Yang K., Huang X., He J., Jiang P. (2015). Strawberry-like Core-Shell Ag@Polydopamine@BaTiO3Hybrid Nanoparticles for High-kPolymer Nanocomposites with High Energy Density and Low Dielectric Loss. Adv. Mater. Interfaces.

[25] Zhao M., Fu Q., Hou Y., Luo L., Li W. (2019). BaTiO3/MWNTs/Polyvinylidene Fluoride Ternary Dielectric Composites with Excellent Dielectric Property, High Breakdown Strength, and High-Energy Storage Density. ACS Omega.

