# Supplementary material for: Construction of a Three-Dimensional BaTiO3 Network for Enhanced Permittivity and Energy Storage of PVDF Composites
Source: Materials (Basel). 2021 Jun 27;14(13):3585. doi: 10.3390/ma14133585 (PMC8269637; doi:10.3390/ma14133585)
Supplement: Supplementary file 1 [file materials-14-03585-s001.zip › materials-1250658-supplementary.pdf]

materials

Supplementary Information

Construction of a Three-Dimensional $BaTiO_3$ Network for Enhanced Permittivity and Energy Storage of PVDF Composites

Xueqing Bi [1], Lujia Yang [1], Zhen Wang [1], Yanhu Zhan [1], Shuangshuang Wang [1], Chunmei Zhang [1], Yuchao Li [1,*], Yinggang Miao [2] and Junwei Zha [3]

[1] School of Materials Science and Engineering, Liaocheng University, Liaocheng 252059, China; bixueqing@gmail.com (X.B.); Iujiay0582@yeah.net (L.Y.); 2010180105@stu.lcu.edu.cn (Z.W.); zhanyanhu@lcu.edu.cn (Y.Z.); wangshuangshuang@lcu.edu.cn (S.W.); zhangchunmei@lcu.edu.cn (C.Z.)
[2] Joint International Research Laboratory of Impact Dynamics and its Engineering Applications, School of Aeronautics, Northwestern Polytechnical University, Xi'an 710072, China; miaoyinggang@nwpu.edu.cn
[3] School of Chemistry and Biological Engineering, University of Science and Technology Beijing, Beijing 100083, China; zhajw@ustb.edu.cn
* Correspondence: liyuchao@lcu.edu.cn; Tel.: +86-635-8230936

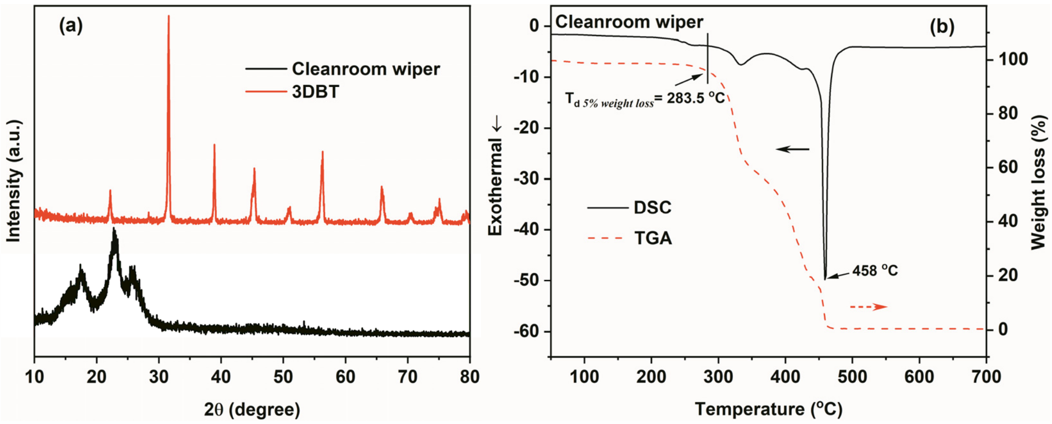

Figure S1. (**a**) XRD result and (**b**) Thermal performance of cleanroom wiper.

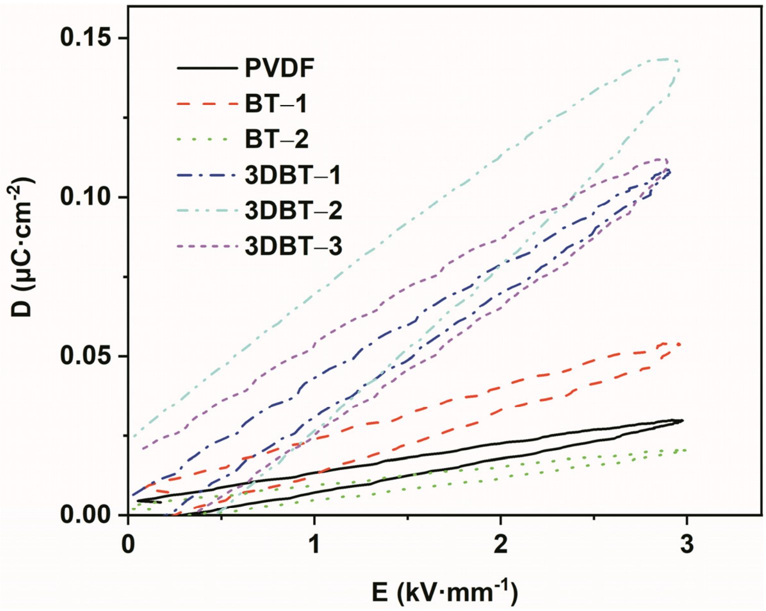

Figure S2. Unipolar D-E hysteresis loops of representative PVDF, BT-1, BT-2, 3DBT-1, 3DBT-2, and 3DBT-3, respectively.

Table S1. Dielectric and energy storage properties of the BT/polymer composites.

Matrix	Filler	Filler loading	Thickness	*f* (Hz)	ε′	Tanδ	E_b (Weillbull)	U	U/U_m	Ref.
PVDF	BST	40 vol%	–	1 kHz	40	0.19	45 kV·mm^{-1}	0.36 J·cm^{-3} (at 1 kV·mm^{-1})	<2.0	[6]
Epoxy	3D BT	16 vol%	0.5-1 μm	1 kHz	34.5	0.07	489.35 kV·cm^{-1}	8.3 mJ·cm^{-3} (at 100 kV·cm^{-1})	16.6	[10]
Epoxy	3D-BTO	10 vol%	1 mm	1 MHz	42	0.09	–	1.57 mJ·cm^{-3} (at 25 kV·cm^{-1})	1.45	[19]
P(VDF-HFP)	BT@Ag	20 vol%	30–40 μm	0.1 Hz	37.5	0.143	247.7 kV·mm^{-1}	3.21 J·cm^{-3} (at 140 kV·mm^{-1})	~3.2	[24]
P(VDF-HFP)	BT	20 vol%	30–40 μm	0.1 Hz	47	0.382	145.1 kV·mm^{-1}	2.45 J·cm^{-3} (at 140 kV·mm^{-1})	~1.4	[24]
PVDF	BT@BN	5 wt%	5 μm	1 kHz	11.3	0.027	580 kV·mm^{-1}	17.6 J·cm^{-3} (at 600 kV·mm^{-1})	2.8	S1
PVDF-epoxy	3D BT	25 vol%	0.1–1μm	1 kHz	200	0.07	322 kV·cm^{-1}	15.7 mJ·cm^{-3} (at 100 kV·cm^{-1})	3.48	S2
PVDF	PHEMA@BT	20 vol%	30–50 μm	1 kHz	20.2	0.03	~140 kV·mm^{-1}	2.18 J·cm^{-3} (at 140 kV·mm^{-1})	~1.1	S3
PVDF	BT@Ni	3 vol%	10–15 μm	1 kHz	12.57	0.027	369 kV·mm^{-1}	9.55 J·cm^{-3} (at 350 kV·mm^{-1})	2.08	S4
PVDF	3D BT	7.5 vol%	~130 μm	100 Hz	25.3	0.057	73.8 kV·mm^{-1}	1.60 mJ·cm^{-3} (at 3 kV·mm^{-1})	4.47	This work

Note: BST= barium strontium titanate; PHEMA = poly(hydroxyethyl methacrylate); U_m is the energy density of pure matrix.

References

S1. S. Luo, J. Yu, S. Yu, R. Sun, L. Cao, W. Liao, C. Wong, Significantly enhanced electrostatic energy storage performance of flexible polymer composites by introducing highly insulating-ferroelectric microhybrids as fillers, Adv. Energy Mater. 9 (2019) 1803204. DOI: 10.1002/aenm.201803204.

S2. Z. Zhang, S. Luo, S. Yu, Z. Guan, R. Sun, C. Wong, Significantly enhanced dielectric and energy storage performance of blend polymer-based composites containing inorganic 3D-network. Mater. Design 142 (2018) 106-113. DOI: 10.1016/j.matdes.2018.01.009.

S3. M. Zhu, X. Huang, K. Yang, X. Zhai, J. Zhang, J. He, P. Jiang, Energy storage in ferroelectric polymer nanocomposites filled with core-shell structured polymer@BaTiO3 nanoparticles: understanding the role of polymer shells in the interfacial regions. ACS appl. Mater. Inter. 6 (2014) 19644-19654. DOI: 10.1021/am504428u.

S4. G. Chen, X. Lin, J. Li, S. Huang, X. Cheng, Core-satellite ultra-small hybrid Ni@BT nanoparticles: A new route to enhanced energy storage capability of PVDF based nanocomposites. Appl. Surf. Sci. 513 (2020) 145877. DOI: 10.1016/j.apsusc.2020.145877.
